# Modeling of solid oxide fuel cells and optimal parameter extraction at various operating data using an optimization method

**DOI:** 10.1371/journal.pone.0350332

**Published:** 2026-06-02

**Authors:** Amlak Abaza, Ragab A. El-Sehiemy, Rania M. Ghoniem, Mahana M. Elbana, Ahmed Bayoumi

**Affiliations:** 1 Electrical Engineering Department, Faculty of Engineering, Kafrelsheikh University, Kafrelsheikh, Egypt; 2 Széchenyi István University, Sustainability Competence Centre, Egyetem tér 1, Győr, Hungary; 3 Department of Information Technology, College of Computer and Information Sciences, Princess Nourah bint Abdulrahman University, Riyadh, Saudi Arabia; 4 Physics and Engineering Mathematics Department, Faculty of Engineering, Kafrelsheikh University, Kafrelsheikh, Egypt; Indian Institute of Technology Guwahati, INDIA

## Abstract

One promising technology for a clean and effective energy conversion option is the solid oxide fuel cell (SOFC) being developed for a broad, widespread role in mobile equipment power supply, and stationary power generation. In this endeavor, an optimal design model based on extracted unknown parameters of the SOFC stack, a dimensional nonlinear optimization problem, is developed using the Puma optimization algorithm (POA). The idea of predator-prey relationships in the natural world forms the basis of POA. By implementing innovative and powerful techniques at every stage of exploration and exploitation, this algorithm has enhanced its performance against a broad variety of optimization tasks. Additionally, a new class of intelligent mechanisms, which is a type of phase change hyper-heuristic, is proposed. There are four operating circumstances in which the stack model is tested: four temperatures in the range 923–1073 K and 3 bar, with two conditions for validation and the others for testing the model. The proposed POA is compared with several well-known algorithms. The findings of the simulation are contrasted with those from published works using the Marine Predator Algorithm (MPA), Moth Flame Algorithm (MFA), Sine Cosine Algorithm (SCA), and Grey Wolf Optimizer (GWO), demonstrating the superior performance of POA in comparison to these competitive algorithms. Under different operating conditions, the computed polarization curves, V-I and P-I, closely resemble the measured datasets. Statistical indices and the ANOVA test confirm that there are differences in the mean values among the optimizer groups, demonstrating the viability and robustness of the proposed optimizer in comparison to other recent complex optimizers. Finally, the proposed POA yields significantly improved parameters with good convergence rates across various SOFC operating conditions.

## 1. Introduction

In the area of energy storage and conversion, fuel cells have shown great promise [1]. They provide a reliable and efficient substitute for conventional combustion-based power plants [2]. Fuel cell is a powerful device used to extract electrical energy directly from chemical oxidation reaction. Such device is composed of an electrolyte, a cathode, and an anode. Anode and cathode aid in the electrochemical reactions, whereas the electrolyte serves as a conduit for the ion’s motion. There exist a lot of fuel cell types classified according to the type of electrolyte that is employed, such as molten carbonate fuel cells (MCFCs) [3], alkaline fuel cells (AFCs) [4], proton exchange membrane (PEM) fuel cells [5,6], and solid oxide fuel cells (SOFCs) [7].

High temperatures (usually exceeding 600°C) are required for SOFC operation, and a range of fuels, including hydrogen, natural gas, and gas generated from coal, can be used. They can be used for combined heat and power applications and are appropriate for stationary power generation systems [8–12].

In recent times, the SOFCs have drawn a lot of interest as a potentially effective and clean technology of energy. The performance of SOFCs is highly dependent on various parameters, such as the operating temperature, fuel composition, and cell geometry [13]. Accurate estimation of these parameters is crucial for optimizing efficiency and performance of SOFCs. However, due to the complex nature of the fuel cell system, traditional optimization methods often face challenges in achieving reliable parameter estimation [14–16].

An alternative method for estimating the parameters of SOFCs is offered by using metaheuristic optimization methods. Recently, metaheuristic optimization techniques have become effective resources for resolving challenging optimization issues [17]. These algorithms can effectively traverse the search space to identify optimal or nearly optimal solutions. They draw inspiration from natural events [18]. Numerous optimization issues, including parameter estimates in diverse domains, have been effectively solved using metaheuristic algorithms including simulated annealing [19], particle swarm optimization algorithm [20], and genetic algorithm (GA) [21]. Reference [22] presents an inverse modeling-based method for estimating thermophysical properties of fouling layers. To solve this inverse problem, the GA was developed to handle the complex and non-linear nature of parameter estimation [23].

There are a lot of work to estimate the SOFCs parameters using metaheuristic methods such as; Utilizing the enhanced chaotic GWO algorithm as a creative and effective way for evaluating the parameters of the solid oxide fuel cell model with good approximation [24], Bald eagle search optimizer-based optimal parameter estimate of the solid oxide fuel cell model [25], Modern optimization techniques for solid oxide fuel cell optimal parameter estimates [26], Finding the SOFC model’s optimal parameters with a modified Gray-Woolf Optimization Algorithm [27], Estimating SOFC model parameters with a modified Cat Optimization Algorithm [28], An approach utilizing political optimizers to estimate optimal parameters for SOFC in both static and dynamic models [29], Artificial ecosystem optimizer [30], machine learning-based metaheuristic [31], atom search optimizer [32], bald eagle search optimizer [25], biogeography-based optimization algorithm [33], coyote optimization algorithm [34], enhanced efficient optimization algorithm (EINFO) [35], JAYA algorithm and Nelder-Mead simplex [36], Harris Hawks optimization technique [37], whale optimization algorithm [38], Tree growth algorithm [39], pathfinder algorithm [40], Differential Evolution [41], Marine predators and political optimizers [42], grasshopper optimizer [43], the Northern Goshawk Optimization (NGO) algorithm [44], Parameter adaptive SCA algorithm [45], and educational competition optimizer (ECO) in [46]. Additionally, in the case of PEM fuel cells, a dynamic ant colony optimization (DACO) algorithm has been developed for accurate parameter estimation and had better convergence and less SSE compared to other metaheuristic methods [47], and in the case of the polymer electrolyte fuel cell (PEFC), A quantitative dual-layer cathode model was constructed to identify the ideal parameters that reduce the over-potential difference and enhance efficiency, utilizing a novel variable-population bat swarm algorithm integrated with computational intelligence-aided design [48].

An innovative method of optimization technique, referred to as the Puma Optimization Algorithm (POA) has demonstrated promising performance in a number of optimization tasks. The idea of predator-prey relationships in the natural world forms the basis of POA. This algorithm has enhanced its performance across various optimization problems by introducing novel and powerful methods at each phase of exploration and exploitation. Furthermore, a novel class of intelligent mechanism is introduced, namely a kind of phase change hyper-heuristic. The POA method can balance both phases and carry out a phase change during the optimization process by using this mechanism. Every phase is automatically adjusted according to the nature of the issue [49].

There are various benefits to using the Puma optimizer for parameter estimation of SOFCs. First of all, the parameter estimation problem in SOFCs is high-dimensional and nonlinear, which POA can manage well. Better parameter estimations and increased SOFC performance may result from the algorithm’s capacity to explore the search space and break free from local optima however, Knowing the parameters accurately make the multi stack connection and the integration of SOFC into the powered system better matching and more efficient. Because of the algorithm’s resilience to data mistakes and uncertainties, more precise parameter estimations may be made, improving SOFC behavior management and prediction. Furthermore, POA’s computing efficiency makes it a good choice for controlling and estimating SOFC parameters in real time. The key contribution issues of the current work are:

- proposing a new SOFC model based on the POA method.
- Different operating conditions have been considered in the parameters’ extraction.
- The simulation outcomes are compared with a set of those published in the literature.
- Statistical indices demonstrate the outstanding power of the proposed POA.

Following is the rest section of the current study that include the problem formulation in section 2, the introduced POA is detailed in section 3, Section 4 describes the numerical applications. Section 5 discusses the algorithmic features explaining the performance of POA. Section 6 presents the sensitivity analysis of the tested fuel cell. Section 7 provides a comprehensive discussion of the results. Finally, Section 8 concludes the paper findings.

## 2. Mathematical representation for SOFC

The Solid Oxide Fuel Cell (SOFC) is fueled with hydrogen and oxidized with oxygen. The high-temperature cell is in the vicinity of 600 and 800°C with newer technology in SOFC. The three major components are oxide ion conduction electrolyte, anode as a fuel electrode, and a cathode as oxidant. The reactions in the SOFC are as under [50,51]:


$$\begin{array}{c} Anode\;:\;H_{\text{2}}+O^{\text{2}-}\;\to H_{\text{2}}O+\text{2}e^{-} \end{array}$$ (1)

$$\begin{array}{c} Cathode:\text{ ½}O_{\text{2}}+\text{2}e^{-}\;\to O^{\text{2}-} \end{array}$$ (2)

$$\begin{array}{c} Overall\;reaction\;:\;H_{\text{2}}+\text{½}O_{\text{2}}\to H_{\text{2}}O \end{array}$$ (3)

Negative ions are created at the cathode results from the reduction of the oxygen, which migrate through the ionic conductive electrolyte, that prevents the flow of electrons. At the anode, hydrogen reacts with the oxygen ions that have passed through, generating water and releasing electrons. These electrons then flow through the external electrical loads.

The output voltage of a SOFC, denoted as V_c_, could be determined utilizing the potential of thermodynamics, E_N_, which arises from no load chemical reaction, along with all voltage losses that occur while the conversion procedure is underway [52–54].


$$\begin{array}{c} V_{c}=E_{N}-\left(V_{act}+V_{oh}+V_{conc}\right) \end{array}$$ (4)

Nernst equation for hydrogen/oxygen fuel cells is used to calculate the thermodynamic potential E_N_, taking into account the operating conditions, including temperature (T in Kelvin) and the partial pressures of oxygen, hydrogen, and water ($\overset{-}{\underset{O_{\text{2}}}{P}},\overset{-}{\underset{H_{\text{2}}}{P}}and\;\overset{-}{\underset{H_{\text{2}}O}{P}};\;$ respectively), as follows [54–58]:


$$\begin{array}{c} E_{N}=E_{rs}+\frac{RT}{\text{2}F}\text{ ln}[\frac{\overset{-}{\underset{H_{\text{2}}}{P}}\times {\left(\overset{-}{\underset{O_{\text{2}}}{P}}\right)}^{\text{0}.\text{5}}}{\overset{-}{\underset{H_{\text{2}}O}{P}}}] \end{array}$$ (5)

where $E_{rs}$ refers to reversible standard potential, F stands for constant of Faraday (96485 C/mol), and R is the universal gas constant (8.3145 J/mol/K).

The losses related to SOFC can be categorized into three types: activation and ohmic resistance loss, and concentration over potentials. Both the loading and operating circumstances have an impact on these losses. Energy barriers must be broken before a chemical reaction can start, which causes activation loss to occur at the beginning of the process. The activation voltage loss ($V_{act}$) is explained by the Butler–Volmer equation as follows [59]:


$$\begin{array}{c} V_{act}=A\;{sinh}^{-\text{1}}\left[\left(\frac{J_{L}}{\text{2}J_{o,a}}\right)+\left(\frac{J_{L}}{\text{2}J_{o,c}}\right)\right] \end{array}$$ (6)

where $J_{o,c},\;J_{o,a}\;andJ_{L}\;$ represent the cathode exchange, anode exchange, and load current densities, respectively, measured in mA/cm². A denotes the slope of the Tafel line.

The ohmic voltage loss, $V_{oh}$, caused by the resistance of ions movement through the electrolyte and the resistance to the flow of electrons through the fuel cell’s electrodes. This voltage loss is calculated as the product of the ionic resistance in k Ω cm², $R_{oh}$, and load current density, as detailed in equation (7).


$$\begin{array}{c} V_{oh}=J_{L}R_{oh} \end{array}$$ (7)

The concentration voltage loss, V_conc_, reflects concentration gradients established in the reaction. It is a result of the cell channels’ mass transfer to reaction sites. The loss results because the current density is near the maximum current density, J_m_.


$$\begin{array}{c} V\_conc=-b\times \text{ln}(\frac{\text{1}-J_{L}}{J_{m}})\; \end{array}$$ (8)

In this context, b is a parametric coefficient viewed as a control variable that varies based on the cell’s operational circumstances and can be estimated optimally using a suitable optimizer.

The total output voltage of the SOFC (per cell) is determined using equation (4).

The stack voltage of $N_{c}$ units’ series connected could be determined using equation (9), assuming that the units behave identically.


$$\begin{array}{c} V_{stack}=N_{c}\times V_{c} \end{array}$$ (9)

## 3. Puma Optimization Algorithm (POA)

A mathematical model for the POA, which is based on natural hunting behaviors, is constructed using the fundamental concepts of pumas in the wild and is given and explained. Up to the authors’ knowledge it is the first time for a method to offer an innovative and deliberate phase shift technique that permits phase switching between exploration and exploitation. Nevertheless, during the exploration and exploitation stages, two different approaches have been used to carry out optimization procedures.

The optimization space is likened to the territory of a male puma, and the optimal solution in the POA is compared to the puma’s territory. The female puma represents the other solutions (X_i_). Every solution in this algorithm enters the exploitation or exploration phases in each iteration by applying the mechanism for phases changes. The phases have been carefully and thoughtfully selected. Every exploration step involved a different optimization strategy, and every phase involved two different processes, all of which were inspired by the pumas’ natural environment. The POA has been performed using MATLAB [49] and checked by many mile stone and then was examined by a function implemented also using MATLAB to represent the problem of this work.

### 3.1. Intelligence-based Phase Transition in Puma

Pumas are extremely intelligent animals with great recall. They often hunt in regions where hunting is more common due to their past experiences. It might go to locations where he has previously gone hunting and covered up his kill on his focused hunting expeditions, or they might send him to the latest site where he hasn’t gone hunting in previous stages. We have included both the exploitation stage (regarding puma travels to previously optimistic locations) and the discovery phase (for visiting new locations). POA was driven to change the phases by a unique and ingenious system, as well as the pumas’ memory and intelligence.

The proposed method’s stage change strategy is a kind of heuristic chosen algorithm that makes use of two elements: intensification and diversity, to deliver reward and punishment scoring operations. The phase transition portion was inspired by the intelligence of cougars. There are two schools of thought on this: the first contends that because cougars lack the energy and experience to thoroughly examine new territory for prey, they try to concurrently seek prey. They ambush in potentially productive regions; the section on the unskilled first generation addresses this.

### 3.2. Unexperienced phase

During the initial three iterations, Operations for POA exploration and exploitation are carried out simultaneously until the phase transition phase’s startup is finished. After the third iteration is finished, each phase will identify solutions that were independently produced and greater than the population as a whole. Only the top solutions from the entire set of solutions created are similar to the whole population that takes the place of the existing ones in order to address this issue. At the conclusion of the third iteration, this is accomplished by calculating the overall cost of the solutions generated during both phases.

### 3.3. Experienced phase

Pumas concludes that it makes sense to decide to change phases after three generations. They choose just one stage for the optimization process as they go through more iterations. In this stage, three different functions f1, f2, and f3 are employed to score however these three functions calculated by factorizing the relation between old and new fitness functions in relation with iteration order and the phase [49]. The escalation component is identified in the first function, which also determines which of the two exploration and exploitation stages has been selected and carried out more effectively than the other. In the first function, the exploration phase is given more weight.

### 3.4. Exploration

In the exploration section, these puma-related behaviors have inspired us to search for food. Pumas now randomly explore their area in search of food or randomly approach other pumas and take advantage of their prey. Consequently, the puma will sometimes jump into search area or forage for food in the space that separates them. Prior to refining its solutions during the exploration stage, Puma arranges the population in ascending order. In Eq. 10, dimensions (R_Dim_) are defined as randomly generated values ranging from 0 to 1. rand_1_ is the name of another produced integer number randomly from 0 to 1. In the complete population, solutions X_a,G_, X_b,G_, X_c,G_, X_d,G_, X_e,G_, and X_f,G_ are those in which faces are selected at random. Using Eq. (11), G is also calculated, and rand_2_ is a random number with a uniform distribution that is created from 0 to 1. Equation (12) states that based on the situation, one of the two equations is chosen for produce a various solution. Then, the new solution is applied to improve the previous one.


$$\begin{array}{c} Z_{i,G}=\begin{array}{cc} R_{Dim}*(Ub-Lb)+Lb & \text{If }{rand}_{\text{1}}\;>\;\text{0}.\text{5}\\ X_{a,G}+G\cdot \left(X_{a,G}-X_{b,G}\right)+G\cdot \left(\begin{array}{c} \left(\left(X_{a,G}-X_{b,G}\right)-\left(X_{c,G}-X_{d,G}\right)\right)\\ +\left(\left(X_{c,G}-X_{d,G}\right)-\left(X_{e,G}-X_{f,G}\right)\right) \end{array}\right) & otherwise \end{array}\; \end{array}$$ (10)

$$\begin{array}{c} G=\text{2}\cdot {rand}_{\text{2}}-\text{1} \end{array}$$ (11)

$$\begin{array}{c} X_{new}=\left\{ \begin{array}{c} @lZ_{i,G}\;,\text{ if}(\text{ j}={\text{j }}_{rand\;})\;or\;(\;\leq U){rand}_{\text{3}}\\ X_{a,G},\;otherwise \end{array}\right.\; \end{array}$$ (12)

The answer generated by Eq. (10) is given by Z_i,G_ in Eq. (12). A random number called j_rand_ is generated in relation to some facets of the problem. Similarly, rand_3_ is a generated integer randomly from 0 to 1 using a uniform distribution. To compute NC, use Equation (13). Prior to the optimization procedure, an integer between 0 and 1 was supplied for the parameter U. On the basis of the criterion in Eq. (15), the dimensions number that are substituted with new solutions in each iteration is increased using Eqs. (13–15). In Eq. (14), Npop is overall number of pumas. The enhancement of the solution is controlled by the state in Eq. (15); the dimensions of the solutions are modified only after this state is met.


$$\begin{array}{c} NC=\text{1}-U \end{array}$$ (13)

$$\begin{array}{c} p=\frac{NC}{Npop}\; \end{array}$$ (14)

$$\begin{array}{c} if\;{CostX}_{new}\;<\;{CostX}_{i}\;,\;U=U+p \end{array}$$ (15)

This activity avoids the local optimum and produces a good diversity of product solutions. Nonetheless, the procedure outlined in the exploration phase considers the fact that search agents are ranked by cost at the beginning of each exploration stage iteration, with high-quality answers ranking first and the Eqs. (13–15) in second. given that the value of U parameter is small in the beginning, quality solutions do not change substantially. Subsequently, as this parameter rises, the solutions with higher cost values undergo a range of changes. In order to find significant optimal points, this technique encourages the investigation of less attractive solutions within the problem domain. It is important to note that Eq. (15) will not be applied if the production pumas are not better than the ones already in use, as this would remove the need for additional duplicate discoveries. Nonetheless, the only changes made to high-quality solutions are to avoid the local optimality trap. Lastly, the current solution is used in place of the freshly developed solutions by Eq. (16).


$$\begin{array}{c} {X_{a,G}=X_{new},\;if\;X}_{i,\;new}<\;X_{a,G} \end{array}$$ (16)

The current solution is replaced with the new production solution, Eq. (16), if it is less expensive than the current solution.

### 3.5. Exploitation

The POA uses two distinct operators in the exploitation step to enhance the responses; these two procedures are modeled after the two hunting techniques used by pumas: running and hunting via ambush. In nature, pumas hide amid bushes, trees, or rocks in an attempt to ambush their prey. It sometimes follows its victim, a behavior that can be mimicked with Eq. (17).


$$\begin{array}{c} X_{new}=\left\{ \begin{array}{c} \;{if\;{rand}_{\text{4}}\geq \text{0}.\text{5},\;X}_{new}=\frac{\left(\frac{mean({Sol}_{total})}{Npop}\right)\cdot \overset{r}{\underset{\text{1}}{X}}-(-\text{1}{)}^{\beta }\times X_{i}}{\text{1}+\left(\alpha \cdot {rand}_{\text{5}}\right)}\;\\ otherwise,\;if\;{rand}_{\text{6}}\geq L,\;X_{new}={Puma}_{male}+\left(\text{2}\cdot {rand}_{\text{7}}\right)\cdot exp\left({randn}_{\text{1}}\right)\cdot \overset{r}{\underset{\text{2}}{X}}-X_{i}\\ otherwise,\;X_{new}=\;\left(\text{2}\times {rand}_{\text{8}}\right)\times \frac{\left(F_{\text{1}}\cdot R\cdot X(i)\;+\;F\text{2}\cdot (\text{1}-R)\cdot {Puma}_{male}\right)}{\left(\text{2}\cdot {rand}_{\text{9}}-\text{1}\;+\;{randn}_{\text{2}}\right)}-{Puma}_{male}\; \end{array}\right.\; \end{array}$$ (17)

Equation (17) lists the two strategies used in the POA. Considering that instance 1 in Eq. (17) is used in pumas for sprinting and ambush techniques in the exploitation stage of hunting, and this operation is carried out by a division to imitate pumas’ swift sprint at prey. On the basis of Eq. (17) If rand_5_, a generated number randomly in a uniform distribution of 0–1, is greater than 0.5, the fast-running approach is used. If not, the ambush method is selected, which entails two distinct operations: the first simulates pumas jumping short distances towards other pumas’ hunts, while the second involves long jumps towards the most skilled puma. As stated by Eq. (17) the mean represents the mean function, the overall of all solutions is Sol_total_, and Npop is the overall number of populations required to complete the procedure. $\overset{r}{\underset{\text{1}}{X}}$ is a solution randomly chosen for the entire population, and β is a randomly created (0 or 1). Furthermore, X_i_ is the solution of the above iteration, and L and α are static parameters and need updating ahead of the process of optimization. The optimal population choice is puma_male_, and the randomly produced numbers in the range of 0 and 1 are rand_4_, rand_5_, rand_6_, rand_7_, rand_8_, and rand_9_. In addition, exp is a symbol of the exponential function. $\overset{r}{\underset{\text{2}}{X}}$ is a randomly produced solution and is produced in accordance with Eq. (18), and randn_1_ and randn_2_ are randomly produced numbers in the normal distribution and in the issue dimensions.


$$\begin{array}{c} ound(\text{1}+\;(Npop-\text{1})\cdot \;{rand}_{\text{10}}) \end{array}$$ (18)

In Eq. (18), where rand_10_ is a randomly chosen integer in the range of 0 and 1, and Npop is the Pumas net number, each element of X is rounded to the nearest integer.

Finally, R, F_1_, and F_2_ are determined, correspondingly, using Equations (19–21).


$$\begin{array}{c} R\;=\;\text{2}\cdot {rand}_{\text{11}}-\text{1} \end{array}$$ (19)

$$\begin{array}{c} F_{\text{1}}={randn}_{\text{3}}\cdot exp\left(\text{2}-Iter\cdot \left(\frac{\text{2}}{MaxIter}\right)\right) \end{array}$$ (20)

In the issue dimensions and the normal distribution in Eq. (20), Randn_2_ is a random number. While Max_Iter_ shows the overall number of iterations required to finish the optimization procedure, Iter represents the number of iterations that have been completed thus far. The symbol exp stands for the exponential function.


$$\begin{array}{c} F_{\text{2}}=\;w\times (v{)}^{\text{2}}\cdot cos\left(\left(\text{2}\times {rand}_{\text{12}}\right)\cdot w\right) \end{array}$$ (21)

$$\begin{array}{c} w={randn}_{\text{4}} \end{array}$$ (22)

$$\begin{array}{c} v={randn}_{\text{5}} \end{array}$$ (23)

**Fig 1:**
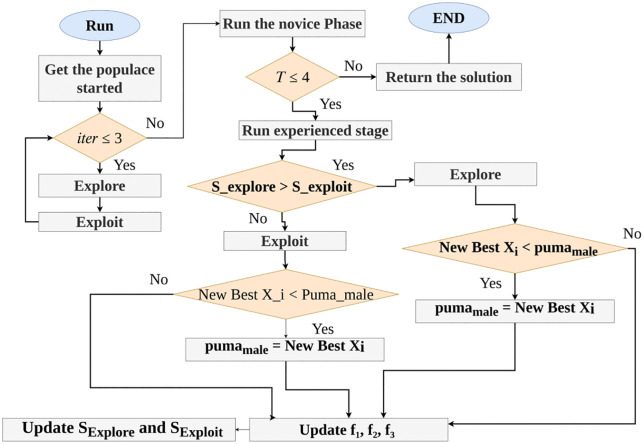
Flowchart of the proposed POA for extracting SOFCs’ Parameters.

Eqs. (21–23) contain both randn_4_ and randn_5_, which are numbers generated randomly in normal distribution and the dimensions of the issue. The number rand_12_ is an integer between 0 and 1 that is created at random, and cos is the symbol for the cosine function. Finally, at the end of this stage, new solutions are replaced if they are less costly than the existing one. The general workflow of the Puma Optimizer (POA) is explained by the flowchart in Fig 1, which notes that T signifies the location of the global best solution and that S_Explore_ and S_Exploit_ reflect the scores for the explore and exploit phases, respectively, according to the functions f_1_, f_2_, and f_3_.

### 3.6. Parameter extraction of SOFC using POA

The goal is to minimize the mean square error (MSE) between voltage estimated by the suggested SOFC stack model and the measured stack voltages, the parameters of the SOFC stack are optimally assessed. To achieve this goal, it is crucial to optimize seven parameters ($E_{rs},\text{ A},{\;J}_{o,a},\;J_{o,c},\;b,\;J_{m}\;and\;R_{oh})$ from equations (4)-(8) that define the proposed model. POA is employed, in this study, to optimize the model’s objective function.


$$\begin{array}{c} MSE=\frac{\text{1}}{n}\sum_{k=\text{1}}^{n}{\left[V_{m}(k)-V_{stack}(k)\right]}^{\text{2}} \end{array}$$ (24)

where, n represents the set points of stack voltages measured $\left(V_{m}\right)\;$ at various load currents. The optimization problem is described as follows:


$$\begin{array}{c} OF=\text{min}(MSE) \end{array}$$ (25)

Subject to:


$$\begin{array}{c} \begin{array}{c} \overset{\text{min}}{\underset{\text{rs}}{\text{E}}}{\leq \text{E}}_{\text{rs}}\leq \overset{\text{max}}{\underset{\text{rs}}{\text{E}}}\;\\ {\text{A}}^{\text{min}}\leq \text{A}\leq {\text{A}}^{\text{max}}\;\\ \begin{array}{c} \overset{min}{\underset{o,i}{J}}\leq J_{o,i}\leq \overset{max}{\underset{o,i}{J}}\;,\;\forall i=a,c\;\\ \begin{array}{c} b^{min}\leq b\leq b^{max}\;\\ \overset{min}{\underset{m}{J}}\leq J_{m}\leq \overset{max}{\underset{m}{J}}\;\\ \overset{\text{min}}{\underset{\text{oh}}{\text{ R}}}{\leq \text{R}}_{\text{oh}}\leq \overset{\text{max}}{\underset{\text{oh}}{\text{R}}} \end{array}\; \end{array}\; \end{array} \end{array}$$ (26)

## 4. Numerical applications

Table 1 presents the characteristics and operating conditions of the tested SOFC stack. The operating data and the technical characteristic of the optimized stack is presented in Table 1 [20]. The min/max operating limits of the control variable of the objective function (OF), as introduced in Eq. (26), is depicted in Table 2. The evaluation of the proposed algorithm was carried out in Ref. [49] on 23 standard functions and CEC2019 functions. These studies proves the effectiveness of the POA compared with several optimization algorithms. In this paper, the parameters of the SOFC stack are extracted by the proposed POA. It is employed to extract these parameters based on the data provided in [25].

**Table 1:** Technical characteristics and operating data of SOFC stack.

Technical characteristics of SOFC stack	Stack parameter	Value
	Stack cells, N_cells_	96 cells
	Rated power (kW)	5 kW
Operating data	P (bar)	3
	Operating Temperature, T (K)	T1 = 923 K, 973 K, 1023 K and 1073 K
	Hydrogen partial pressure	0.9
	Water partial pressure	0.1
	Oxygen partial pressure	0.21
	Reactants	Hydrogen and Air

**Table 2:** Estimated SOFC parameters extracted by POA, MPA, MFO, SCA, and GWO at 3 bar/923 K.

		Optimal parameters							
Bounds		E_0 (V)	A (V)	${𝐉}_{𝐨,𝐚}$ (mA/cm^2^)	${𝐉}_{𝐨,𝐜}$ (mA/cm^2^)	b (V)	${𝐉}_{𝐦}$ (mA/cm^2^)	${𝐑}_{ohm}$ (kW cm^2^)	MSE$\left({𝐕}^{2}\right)$
Lower		0	0	0	0	0	0	0	
Upper		1.2	1	100	100	1	1000	1	
Optimizers	POA	1.1196	0.0596	17.5024	6.2475	0.0528	1.57E + 02	6.1E-03	8.05E-08
	MPA	1.1195	0.0590	17.7393	6.2160	0.0378	1.48E + 02	6.24 E-03	5.55E-05
	MFO	1.1198	0.0585	18.0000	6.0061	0.0476	1.56E + 02	6.19 E-03	1.07E-04
	SCA	1.1186	0.0588	18.0000	7.0000	0.0512	1.57E + 02	6.2 E-03	1.05E-01
	GWO	1.1194	0.0557	17.3751	5.9044	0.0277	1.43E + 02	6.45 E-03	5.15E-04

The extracted values for the seven control variables ($E_{rs},\text{A},J_{o,a},\;J_{o,c},\;b,\;J_{m}\;and\;R_{oh})$ are optimally determined by finding the optimal solution of solving the optimization framework outlined in Eqs. (24) and (26). The obtained parameters are employed to develop a precise model of the SOFCs stack that is tested and validation under four temperature levels of 923, 973, 1023, and 1073 K at a pressure of 3 bar. POA is used to optimally extract the optimal parameters of SOFC (5 kW). The number of populations of the POA is taken as 150 with 500 iterations. The three controlling parameters of POA, U, L and α equal 0.2, 0.67, 2. The simulation results are compared with those from previous works using the Marine Predator Algorithm (MPA) [58], Moth Flame Algorithm (MFA) [60], Sine Cosine Algorithm (SCA) [61,62], and Grey Wolf Optimizer (GWO) [27] showing superior performance of the proposed POA in comparison to competitive algorithms.

Tables 2 and 3 present the outcomes of the simulation of the parameter estimation problem using the introduced POA in comparison to several well-known algorithms, including MPA, MFO, SCA, and GWO. The estimated optimal control variables obtained with the POA are evaluated under different operating conditions (3 bars and temperatures of 923/1073 K).

**Table 3:** Estimated parameters of SOFC extracted by POA, MPA, MFO, SCA, and GWO at 3 bar/1073 K.

		Optimal parameters							
Bounds		E_0 (V)	A (V)	${𝐉}_{𝐨,𝐚}$ (mA/cm^2^)	${𝐉}_{𝐨,𝐜}$ (mA/cm^2^)	b (V)	${𝐉}_{𝐦}$ (mA/cm^2^)	${𝐑}_{ohm}$ (kW cm^2^)	MSE$\left({𝐕}^{2}\right)$
Lower		0	0	0	0	0	0	0	
Upper		1.2	1	100	100	1	1000	1	
Optimizers	POA	1.114805	0.035458	26.38432	6.598204	0.065344	159.8985	4.01 E-03	2.46E-07
	MPA	1.114582	0.0352	27.71667	6.544841	0.064248	159.8579	4.04 E-03	3.56E-04
	MFO	1.114347	0.033787	20.57787	7	0.065927	159.92	4.01 E-03	3.35E-04
	SCA	1.127263	0.047174	21.00065	6.519794	0.094659	162	3.28 E-03	3.89E-01
	GWO	1.116326	0.041673	24.01683	6.90261	0.080284	160.5618	3.59 E-03	4.90E-02

The simulation results in Table 2 confirm that POA outperforms the other competitive algorithms, as ithas enhanced its performance across various optimization problems by introducing novel and powerful methods at each phase of exploration and exploitation. Additionally, POA can dynamically adjust the balance between exploration and exploitation according to specific characteristics of search spaces. This is in contrast to traditional metaheuristics, which often have fixed transition mechanisms.

**Fig 2:**
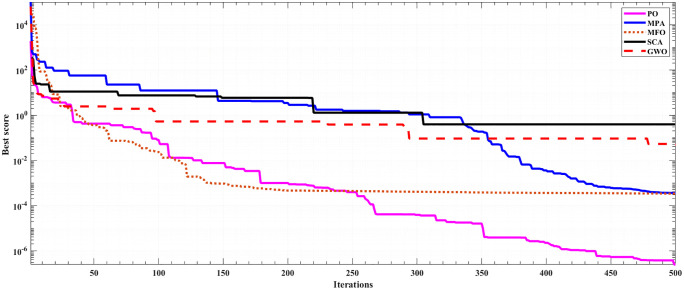
Convergence rate of 5- kW SOFC at 3 bar/ 1073K.

Notably, POA demonstrates the highest accuracy, achieving the lowest mean square error (MSE) values of 8.05E-08 and 2.46E-07at the operating conditions of 3 bar/ 923 K and 3 bar/ 1073 K, respectively. The convergence rates of the proposed POA are illustrated in Fig 2, alongside the other competitive algorithms. It is evident that POA exhibits the best convergence behavior and the lowest MSE among the compared algorithms.

**Fig 3:**
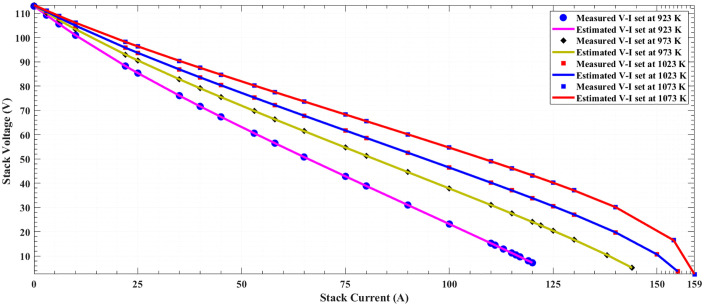
Polarization V-I curves at different operating conditions of SOFC 5-kW stack at 3/923K/973K/1023K/1073K of SOFC 5-kW stack.

**Fig 4:**
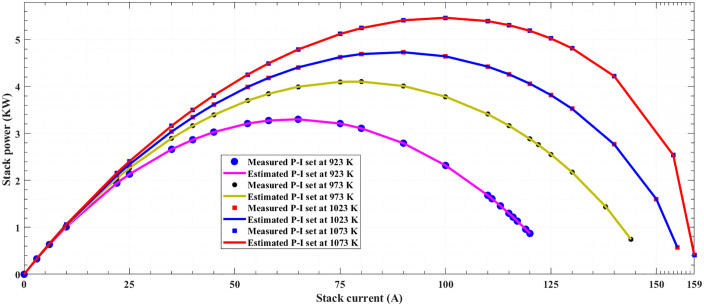
Polarization P-I curves at different operating conditions of SOFC 5-kW stack at 3/923K/973K/1023K/1073K of SOFC 5-kW stack.

The V-I and P-I polarization curves for the 5-kW SOFC stack are displayed respectively in Figs 3 and 4, utilizing both measured and extracted stack voltages at (3 bar and four operating temperatures of 92,/973, 1023, and 1073 K). It is evident that the estimated model aligns well with the measured data across different conditions. This highlights the power of proposed POA in accurately extracting a model for SOFC stack.

The performance and efficiency of POA is assessed through applying the algorithm for 30 times at two test operating temperature 923 K and 1073 K and operating pressure of 3 bars. Statistical indices for the proposed POA, along with the four competitive algorithms (MPA, MFO, SCA, and GWO), are presented in Tables 4 and 5. It is clear that PO yields the best statistical indices. The optimal mean square error (MSE) of 8.05E-08 is achieved by POA, followed by 5.55E-05 from MPA at 923 K. In addition, the lowest MSE is 2.46E-07 via POA followed by 3.35E-04 from MFO at 1073 K. Furthermore, POA demonstrates the best MSE, variance, median, and standard deviation at the two operating temperatures (923 K and 1073 K). These results strongly indicate that POA outperforms the other competitive algorithms.

**Table 4:** Statistical indices of POA, MPA, MFO, SCA, and GWO at 3 bar/923 K (30 runs).

Metric	POA	MPA	MFO	SCA	GWO
Min	8.050E-08	5.550E-05	1.070E-04	1.050E-01	5.150E-04
Max.	6.750E-04	3.910E-01	8.570E-01	2.220E + 00	8.220E-01
Mean	8.620E-05	5.150E-02	1.090E-01	6.840E-01	1.050E-01
Variance	2.230E-08	9.830E-03	4.920E-02	3.060E-01	4.250E-02
Median	1.210E-05	2.210E-03	1.100E-02	4.710E-01	2.500E-02
STD	1.490E-04	9.910E-02	2.220E-01	5.530E-01	2.060E-01

**Table 5:** Statistical indices of POA, MPA, MFO, SCA, and GWO at 3 bar/1073 K (30 runs).

Metric	POA	MPA	MFO	SCA	GWO
Min	2.460E-07	3.560E-04	3.350E-04	3.890E-01	4.900E-02
Max.	1.290E-04	2.230E-01	2.760E + 00	3.840E + 00	2.220E-01
Mean	1.270E-05	7.290E-02	2.160E-01	1.420E + 00	1.320E-01
Variance	5.370E-10	2.470E-03	2.400E-01	5.530E-01	1.980E-03
Median	4.830E-06	7.360E-02	2.060E-01	1.330E + 00	1.310E-01
STD	2.320E-05	4.970E-02	4.900E-01	7.440E-01	4.450E-02

**Fig 5:**
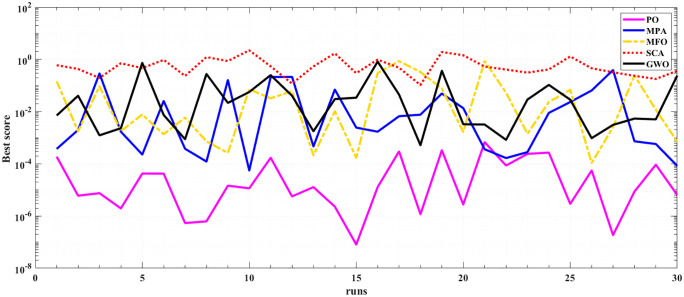
Robustness of PO, MPA, MFO, SCA and GWO at 3 bar/923K.

**Fig 6:**
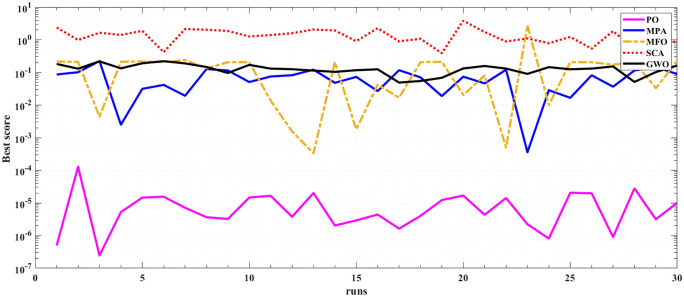
Robustness of PO, MPA, MFO, SCA and GWO at 3 bar/1073K.

**Fig 7:**
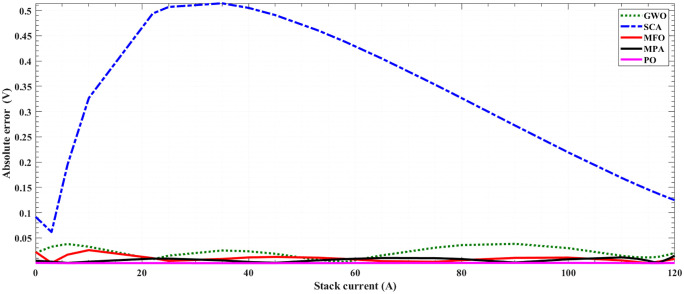
Absolute error for 5-kW SOFC stack at (3 bar/ 923 K) for OP, MPA, MFO, SCA and GWO.

**Fig 8:**
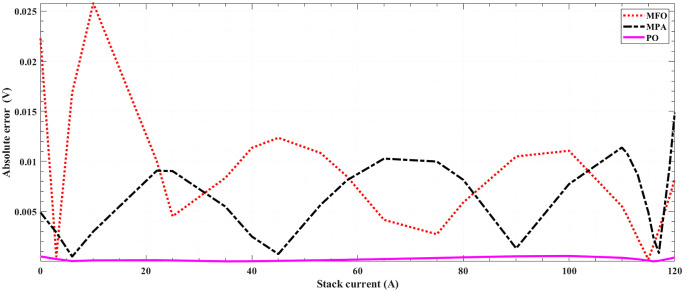
Absolute error for 5-kW SOFC stack at (3 bar/ 923 K) for PO, MPA and MFO.

Figs 5 and 6 illustrate the results of the 30 separate runs at 3 bar/ (923 K and 1073 K). The solution robustness is illustrated through a plot of the fluctuating MSE across these runs. Figs 5 and 6 compare the robustness of POA with various competitive optimizers at 923K and 1073 K. The effectiveness of POA is further highlighted by its robustness characteristics, as it exhibits lower fluctuations compared to MPA and MFO. The absolute stack voltage error (ASVE) of SOFC is one of the best methods used to evaluate the accuracy of the estimated model with the proposed optimization algorithm, POA. Table 6 reports the stack voltage and the corresponding ASVE at 3 bar/923 K operating condition using PO and the other competitive algorithms. It is observed From Figs 7 and 8 that the proposed POA demonstrates the highest accuracy. These findings collectively validate the precision, dominance, and efficiency of the proposed POA in extracting the optimal parameters for the SOFC stack model under the tested operating conditions.

**Table 6:** Simulation results of absolute voltage error for SOFC 5-kW stack at (3 bar/ 923 K).

Optimizer	POA	MPA	MFO	SCA	GWO
${𝐕}_{𝐦}$ (V)	Absolute stack voltage error at 3 bar/923 K (V)				
112.89	5.070E-04	4.900E-03	2.230E-02	9.180E-02	2.050E-02
109.19	2.470E-04	2.910E-03	4.920E-04	6.160E-02	3.270E-02
105.55	4.340E-05	4.970E-04	1.680E-02	1.950E-01	3.790E-02
100.90	1.140E-04	3.000E-03	2.580E-02	3.270E-01	3.260E-02
88.23	1.380E-04	9.070E-03	1.000E-02	4.940E-01	7.730E-03
85.31	1.100E-04	9.030E-03	4.520E-03	5.070E-01	1.500E-02
76.04	1.650E-05	5.480E-03	8.360E-03	5.140E-01	2.520E-02
71.62	2.550E-05	2.500E-03	1.130E-02	5.050E-01	2.350E-02
67.31	6.550E-05	7.470E-04	1.240E-02	4.910E-01	1.860E-02
60.58	1.310E-04	5.660E-03	1.080E-02	4.610E-01	6.450E-03
56.46	1.750E-04	8.120E-03	8.500E-03	4.390E-01	2.590E-03
50.79	2.440E-04	1.030E-02	4.160E-03	4.050E-01	1.540E-02
42.81	3.570E-04	9.980E-03	2.730E-03	3.530E-01	3.070E-02
38.86	4.160E-04	8.150E-03	5.900E-03	3.270E-01	3.570E-02
31.01	5.240E-04	1.320E-03	1.050E-02	2.730E-01	3.830E-02
23.18	5.600E-04	7.730E-03	1.110E-02	2.190E-01	2.980E-02
15.29	3.730E-04	1.140E-02	5.510E-03	1.690E-01	1.480E-02
14.49	3.310E-04	1.080E-02	4.540E-03	1.640E-01	1.360E-02
12.89	2.280E-04	8.650E-03	2.330E-03	1.550E-01	1.180E-02
11.29	9.610E-05	4.930E-03	2.270E-04	1.460E-01	1.130E-02
10.49	1.690E-05	2.330E-03	1.640E-03	1.410E-01	1.170E-02
9.68	7.240E-05	8.540E-04	3.150E-03	1.370E-01	1.280E-02
8.06	2.850E-04	9.350E-03	6.450E-03	1.290E-01	1.710E-02
7.25	4.110E-04	1.490E-02	8.240E-03	1.250E-01	2.070E-02

**Fig 9:**
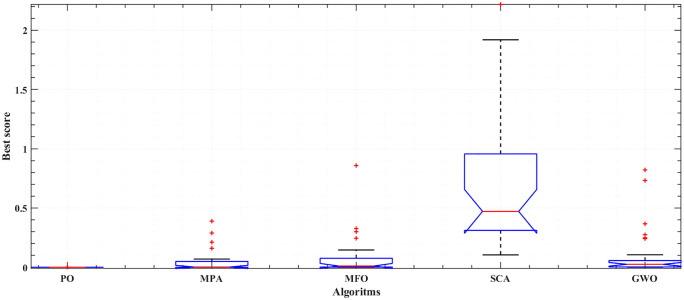
Box plot for the proposed PO compared to MPA, MFO, SCA, and GWO; at 3 bar/923 K.

**Fig 10:**
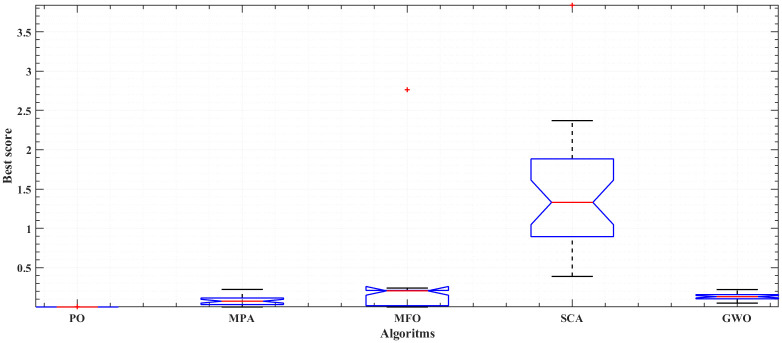
Box plot for the proposed PO compared to MPA, MFO, SCA, and GWO; at 3 bar/1073 K.

The statistical ANOVA test indicates whether there are significant differences in the mean values among the groups of optimizers, including the POA, MPA, SCA, and GWO (at 3 bar/923 K and 3 bar/1073 K). It can be applied using MATLAB functions. A lower ρ value (p < 0.05) suggests the presence of significant variability between the means of the optimizer results. This statistical analysis detailed are summarized in Table 7, highlights that the POA optimizer achieves a lower mean. Additionally, the lower mean achieved by the POA is depicted in the box plots for every optimizer, as illustrated in Figs 9 and 10 for both operating conditions. Overall, the statistical outcomes highlight the efficiency and dominance of the POA compared to other established contending optimizers in assessing an accurate model of SOFC stack at different operating conditions.

**Table 7:** ANOVA test for POA compared to MPA, MFO, SCA, and GWO at 3 bar/923K/1073K.

conditions	Source	SS	df	MS	‘Chi-sq’	ρ>Chi-sq’
**3 bar/923K**	Columns	211.6667	4	52.91667	84.66667	1.79E-17
	Error	88.33333	116	0.761494		
	Total	300	149			
**3 bar/1073K**	Columns	244.9333	4	61.23333	97.97333	2.66E-20
	Error	55.06667	116	0.474713		
	Total	300	149			

## 5. Algorithmic features explaining POA performance

The non-linearity handling: The SOFC voltage model has exponential non-linearity in the activation loss term (Equation 6), logarithmic non-linearity in the Nernst potential term (Equation 5), and hyperbolic non-linearity in the concentration loss term (Equation 8). POA’s dual phase method can be applied to solve this problem: in the unexperienced phase (first 3 iterations), simultaneous exploration and exploitation can avoid convergence to local minima in the exponential activation loss term; in the experienced phase, intelligent scoring functions f₁, f₂, f₃ can be applied for fine-tuning in the logarithmic concentration loss term.

Parameter Interdependence: All seven parameters show high interdependence. For example, changes in reversible potential (E_rs_) directly impact the baseline for activation losses (A, J_o, a_, J_o, c_), which in turn impact ohmic resistance (R_oh_). POA’s phase change mechanism automatically adjusts its search intensity according to parameter sensitivity, as evidenced by the reduction in variance in Tables 8 and 9. While the population drops from 150 to 80, MSE only increases by a factor of 5.6 (2.46 × 10 ⁻ ⁷ to 1.38 × 10 ⁻ ⁶).

**Table 8:** Sensitivity analysis for the estimated parameters of SOFC extracted by POA related to the number of populations at 3 bar/1073 K.

		Optimal parameters							
Bounds		E_0 (V)	A (V)	${𝐉}_{𝐨,𝐚}$ (mA/cm^2^)	${𝐉}_{𝐨,𝐜}$ (mA/cm^2^)	b (V)	${𝐉}_{𝐦}$ (mA/cm^2^)	${𝐑}_{ohm}$ (kW.cm^2^)	MSE$\left({𝐕}^{2}\right)$
Lower		0	0	0	0	0	0	0	
Upper		1.2	1	100	100	1	1000	1	
Populations	150	1.1148	0.0355	26.3843	6.5982	0.0653	159.8985	0.0040	2.46E-07
	120	1.1148	0.0372	29.7001	6.7736	0.0655	159.9019	0.0040	4.094E-07
	100	1.1148	0.0354	26.1953	6.5965	0.0653	159.8981	0.0040	4.034E-07
	80	1.1148	0.0374	29.9603	6.7838	0.0655	159.9040	0.0040	1.3766E-06
	60	1.1148	0.0353	25.9510	6.5880	0.0654	159.8988	0.0040	2.73E-07

**Table 9:** Sensitivity analysis for Estimated parameters of SOFC extracted by POA related to the maximum number of iteration at 3 bar/1073 K.

		Optimal parameters							
Bounds		E_0 (V)	A (V)	${𝐉}_{o,𝐚}$ (mA/cm2)	${𝐉}_{o,𝐜}$ (mA/cm2)	b (V)	${𝐉}_{𝐦}$ (mA/cm2)	${𝐑}_{ohm}$ (kW.cm2)	MSE $\left({𝐕}^{2}\right)$
Lower		0	0	0	0	0	0	0	
Upper		1.2	1	100	100	1	1000	1	
No. of iterations	500	1.1148	0.0355	26.3843	6.5982	0.0653	159.8985	0.0040	2.46E-07
	450	1.1148	0.0350	25.5066	6.5760	0.0653	159.8982	0.0040	4.7268-07
	400	1.1148	0.0360	27.5253	6.6293	0.0654	159.9006	0.0040	2.477E-06
	350	1.1148	0.03401	23.71584	6.49753	0.06521	159.89486	0.00403	2.493E-06
	300	1.1148	0.0374	29.9620	6.7853	0.0655	159.9042	0.0040	4.521E-06
	250	1.1148	0.0359	27.2229	6.6083	0.0654	159.9017	0.0040	1.386E-05
	200	1.1147	0.0358	27.6158	6.6671	0.0647	159.8760	0.0040	2.134E-04
	150	1.1153	0.0332	25.6887	5.8847	0.0641	159.8519	0.0041	9.337E04

Convergence Evidence: From Fig 2, it is evident that POA converges to optimal MSE within 200 iterations. On the other hand, MPA, MFO, SCA, and GWO need 300–500 iterations or fail to converge to a comparable MSE. This is due to the fact that POA incorporates a phase change strategy consisting of fast escape from exploration (iterations 1–50), intelligent phase choice (iterations 50–150), and precise exploitation (iterations 150–200). Competitor algorithms lack this adaptive structure.

## 6. Sensitivity analysis for the tested fuel cell

The sensitivity of POA to the number of populations is testing at 150, 120, 100, 80, and 60 populations. The results are explained in Table 8. The results introduce the estimated parameters of SOFC stack at 3 bar/1073 K. It is clear that MSE value slightly affect by the number of populations, as it is ranges from 2.46E-07 to 1.3766E-06. Similarly, another index for measuring the sensitivity analysis is studying the sensitivity for varied number of iterations as presented in Table 9. The results are carried out at 3 -bar 1073 K. The increase of iteration number from 150 to 500 reduces the MSE from 9.337E-4 to 2.76E-7. Both tables prove the results confirm the quality and the stability of POA.

Table 10 shows Comparison between POA and other optimization algorithms in terms of mean absolute error (MAE) as fitness function, convergence time and the iteration numbers associated with Success rate. As presented in this table, the POA archives the least MAE. Therefore, it indicates an excellent accuracy for parameter estimation in fuel cells compared with other competitive algorithms.

**Table 10:** Evaluation of POA alongside various optimization techniques in terms of the MAE fitness function, convergence time and the iteration numbers associated with Success rate.

Algorithm	MAE	Convergence Time (s)	Number of Iterations	Success Rate (%)
Puma Optimization Algorithm (POA)	0.0021	12.5	200	97
Particle Swarm Optimization [63]	0.0034	18.9	300	85
Genetic Algorithm [64]	0.0048	25.3	350	78
Differential Evolution [65]	0.0030	19.2	300	88
Ant Colony Optimization [66]	0.0062	35.4	500	65
Simulated Annealing [67]	0.0058	40.1	450	68
Grey Wolf Optimizer [68]	0.0024	15.2	250	92
Whale Optimization Algorithm [69]	0.0029	17.8	275	90
Firefly Algorithm [70]	0.0050	30.6	400	70

## 7. Discussion

In this section, authors present the advantages and disadvantage and suggested for future tests prove the capability of POA in the future as: First the Advantages can be stated as follows: Among the simulation results in the revised version, the PUMA algorithm has a fast convergence speed alongside other optimization techniques, has efficient exploration and exploitation phases that leads to more optimal solutions. The sensitivity analysis prove high robustness against local minima. The PUMA algorithm needs few computation times to reach the optimal solutions. The simulation results are tested for 30 different runs that give improved statistical indices compared to other optimization techniques. Secondly the disadvantages are stated as follows: The sensitivity to maximum population size give an impression that the PUMA algorithm is highly sensitive to these parameters as appeared in Tables 8 and 9. The scalability of the suggested algorithm is not tested in proper manner in this study that need to be considered.

## 8. Conclusions

A recent optimizer called the Puma Optimization Algorithm has been applied to find the optimal solution of high non-linear problems that are related to the parameter estimation of the SOFCs stack. These parameters are optimally extracted by taking into account the primary objective function that aims at reducing the mean square error between the voltage estimated by the SOFC stack and the measured stack voltages. To achieve this goal, it is crucial to optimize the seven parameters model. The numerical simulation results of using the introduced POA are compared with several well-known algorithms, including MPA, MFO, SCA, and GWO. The estimated optimal control variables obtained with the POA are assessed under different operating conditions. The simulation results confirm that POA outperforms the other competitive algorithms. Particularly, POA demonstrates the highest accuracy, achieving the lowest mean square error of 8.05E-08 and 2.46E-07 at the operating temperatures 923 K and 1073 K at an operating pressure of 3 bar, respectively. The convergence rates of the proposed POA are evidence that the POA exhibits the best convergence behaviour and the lowest MSE among the compared algorithms. Also, the ANOVA test indicates whether there are significant differences in the mean values among the groups of optimizers, including the POA (at 3 bar/923 K and 3 bar/1073 K). A lower ρ value (p < 0.05) suggests the presence of significant variability between the means of the optimizer results. Added to that, the statistical indices highlight the effectiveness and supremacy of the newly developed POA in contrast to previous well-known rival optimizers in extracting a precise model of SOFC stack at different operating conditions. Future testes for large scale application are needed to prove the high capability of PUMA algorithm with more control variables and for multiobjective frameworks such as in optimal power flow problems.

Ethics approval and consent to participate: Not applicable.

Consent for publication: Not applicable.

## Supporting information

S1 File Supporting Information files. (ZIP)
