# Supplementary material for: Modeling of solid oxide fuel cells and optimal parameter extraction at various operating data using an optimization method
Source: PLoS One. 2026 Jun 2;21(6):e0350332. doi: 10.1371/journal.pone.0350332 (PMC13229311; doi:10.1371/journal.pone.0350332)
Supplement: S1 File — (ZIP) [file pone.0350332.s001.zip › Supporting Information files/data.docx]

% F14

aS=[-32 -16 0 16 32 -32 -16 0 16 32 -32 -16 0 16 32 -32 -16 0 16 32 -32 -16 0 16 32;...

-32 -32 -32 -32 -32 -16 -16 -16 -16 -16 0 0 0 0 0 16 16 16 16 16 32 32 32 32 32];

% F15

aK=[.1957 .1947 .1735 .16 .0844 .0627 .0456 .0342 .0323 .0235 .0246];

bK=[.25 .5 1 2 4 6 8 10 12 14 16];

% F19

aH=[3 10 30;.1 10 35;3 10 30;.1 10 35];

cH=[1 1.2 3 3.2];

pH=[.3689 .117 .2673;.4699 .4387 .747;.1091 .8732 .5547;.03815 .5743 .8828];

% F20

aH=[10 3 17 3.5 1.7 8;.05 10 17 .1 8 14;3 3.5 1.7 10 17 8;17 8 .05 10 .1 14];

cH=[1 1.2 3 3.2];

pH=[.1312 .1696 .5569 .0124 .8283 .5886;.2329 .4135 .8307 .3736 .1004 .9991;...

.2348 .1415 .3522 .2883 .3047 .6650;.4047 .8828 .8732 .5743 .1091 .0381];

% F21

aSH=[4 4 4 4;1 1 1 1;8 8 8 8;6 6 6 6;3 7 3 7;2 9 2 9;5 5 3 3;8 1 8 1;6 2 6 2;7 3.6 7 3.6];

cSH=[.1 .2 .2 .4 .4 .6 .3 .7 .5 .5];

% F22

aSH=[4 4 4 4;1 1 1 1;8 8 8 8;6 6 6 6;3 7 3 7;2 9 2 9;5 5 3 3;8 1 8 1;6 2 6 2;7 3.6 7 3.6];

cSH=[.1 .2 .2 .4 .4 .6 .3 .7 .5 .5];

% F23

aSH=[4 4 4 4;1 1 1 1;8 8 8 8;6 6 6 6;3 7 3 7;2 9 2 9;5 5 3 3;8 1 8 1;6 2 6 2;7 3.6 7 3.6];

cSH=[.1 .2 .2 .4 .4 .6 .3 .7 .5 .5];
